# Effects of Climate Change on Exposure to Coastal Flooding in Latin America and the Caribbean

**DOI:** 10.1371/journal.pone.0133409

**Published:** 2015-07-15

**Authors:** Borja G. Reguero, Iñigo J. Losada, Pedro Díaz-Simal, Fernando J. Méndez, Michael W. Beck

**Affiliations:** 1 Environmental Hydraulics Institute “IH Cantabria”, Universidad de Cantabria, Santander, Cantabria, Spain; 2 Institute of Marine Sciences, University of California Santa Cruz, Santa Cruz, CA, United States of America, and The Nature Conservancy, Arlington, VA, United States of America; Universidade de Vigo, SPAIN

## Abstract

This study considers and compares several of the most important factors contributing to coastal flooding in Latin American and the Caribbean (LAC) while accounting for the variations of these factors with location and time. The study assesses the populations, the land areas and the built capital exposed at present and at the middle and end of the 21^st^ century for a set of scenarios that include both climatic and non-climatic drivers. Climatic drivers include global mean sea level, natural modes of climate variability such as El Niño, natural subsidence, and extreme sea levels resulting from the combination of projected local sea-level rise, storm surges and wave setup. Population is the only human-related driver accounted for in the future. Without adaptation, more than 4 million inhabitants will be exposed to flooding from relative sea-level rise by the end of the century, assuming the 8.5 W m^−2^ trajectory of the Representative Concentration Pathways (RCPs), or RCP8.5. However, the contributions from El Niño events substantially raise the threat in several Pacific-coast countries of the region and sooner than previously anticipated. At the tropical Pacific coastlines, the exposure by the mid-century for an event similar to El Niño 1998 would be comparable to that of the RCP4.5 relative sea-level rise by the end of the century. Furthermore, more than 7.5 million inhabitants, 42,600 km^2^ and built capital valued at 334 billion USD are currently situated at elevations below the 100-year extreme sea level. With sea levels rising and the population increasing, it is estimated that more than 9 million inhabitants will be exposed by the end of the century for either of the RCPs considered. The spatial distribution of exposure and the comparison of scenarios and timeframes can serve as a guide in future adaptation and risk reduction policies in the region.

## Introduction

With increasing effects and greater damage from climate hazards occurring worldwide, there is increasing interest in risk reduction, informed development, resilience and climate adaptation [1–3]. This increased interest is particularly keen in coastal zones, where there has been and will likely continue to be rapid increases in populations, development and, consequently, risk [4–10]. To make matters worse, these increases are appearing concurrently with sharply rising sea levels and changes in extreme sea levels associated with storms (e.g., [11–12]), thereby adding to already greater risks.

Coastal areas with elevations less than 10 m above mean sea level (MSL) account only for 2% of the Earth’s surface but are inhabited by approximately 10% of the population of the earth [6, 13]. Population concentrations in low-elevation areas are even greater in developing countries. It has been estimated that globally between 660,000 and 1,200,000 km^2^ of land, 93–310 million inhabitants and 3,100–11,000 billion USD of built capital are located at elevations less than the present 100-year flooding event [7, 13]. Current estimates increase when climate-induced sea level increases and land subsidence are considered [13, 14]. Furthermore, by mid-century there could be an eight-fold increase in global flood losses for the 136 largest coastal cities based on projected socio-economic changes alone [15].

In Latin America and the Caribbean (LAC), populations continue to increase, particularly in coastal zones, where both intensive development and high levels of poverty already exist. Several cities in LAC currently have high levels of exposure to flooding and many others will experience a significant increase by the middle of the century [15]. Regional estimates for LAC point to between 29 and 32 million people living in the first 10 m of elevation and over 6 million in the 100-yr flood plain for population levels of year 2000 [6, 13]. Furthermore, the region’s economy is heavily dependent on natural resources and tourism [16, 17] and has been shown to be sensitive to meteorological and environmental changes and stresses in the past [18–21]. It is also expected that these effects will be greater with a changing climate [19, 20, 13, 14]. However, there is a lack of information and data to characterize populations and places most at risk from coastal hazards in the region [20], but this information is critical in making present and future planning decisions. An understanding of the drivers of risks in these highly dynamic coastal zones is critical socially and economically.

Sea levels have steadily risen in recent decades and have contributed to increased extreme sea levels at most locations globally [22, 23, 11, 12]; however, sea-level rise (SLR) and its acceleration remain a subject of research and debate for planning and decision making [24]. More importantly, the mean sea level will change both in the long term and on an inter-annual scale because of climate variability patterns, of which El Niño [21] in the Pacific Ocean is one of the most prominent examples [25, 26]. In our analysis, we provide a regional spectrum of present and future exposures of land, populations and built capital for various sea-level rise projections, inter-annual variations (associated with El Niño events), extreme sea levels and population levels. We identify where and to what extent negative effects are to be expected in LAC. The analysis identifies the geographic areas most exposed in LAC on the basis of homogenous principles and data on a continental scale. We consider climatic and non-climatic drivers across a set of scenarios for various timeframes to identify the drivers that may pose the greatest threat to inhabitants and property and the timeframes and the locations at greatest exposure.

## Methods and Data

### 2.1. Definitions of drivers and scenarios

This study accounts for the variations with location and time in the distribution of coastal flooding exposure on a continental scale by considering the contributions of some of the most important drivers of flooding. A description of each driver follows.

#### Regional sea-level rise

The regional sea-level rise data for the period 1950–2011 were obtained from [26], and the projections for the middle (2046 and 2065) and the end of the century (2081–2100), which are based on the CMIP5 climate models for the RCP4.5 and RCP8.5 scenarios, were obtained from [27]. RCP4.5 represents a moderately warming climate, and RCP8.5 represents a warmer climate. Both pathways include contributions from steric and dynamic sea surface heights, atmospheric pressure loading and glacier and polar ice sheet surface mass balances, the dynamic ice sheet contribution, groundwater depletion and the glacial isostatic adjustment. For mid-century, we use a single scenario (RCP4.5) because the differences between the RCPs in this timeframe are relatively small in comparison with those for the second half of the century (the global mean values of sea-level rise for the mid-century are 0.26 m and 0.3 m for RCP4.5 and RCP8.5, respectively) [11].

#### El Niño (ENSO)

Several authors have shown that the inter-annual variability arising from natural modes influences sea levels in the region. For example, [25] calculated that in the equatorial Pacific region, sea levels can deviate from the global mean sea level by as much as 40 cm because of ENSO. In [26], after evaluating the influence of several climate indices on sea level anomalies in the region, the authors concluded that ENSO is the index with the greatest correlation to sea level components. Based on that result, our study assumes that ENSO is the main driver of inter-annual variability for coastal flooding in LAC. The same approach could have been followed for other relevant modes in the area. Finally, it is important to understand that should changes in these climatic modes occur in the future with respect to the present dynamics, a factor that we do not account for in this analysis, these changes will consequently be reflected in the flooding.

Using the data in [26], we evaluated ENSO-induced sea level changes from historical mean sea level records. Because of the uncertainties in the projected frequencies and the intensities of the ENSO events over the century, no change beyond ENSO historical variations was assumed. We also considered the El Niño 1998 event (N_98_) as a representative figure of the contribution of ENSO to flooding levels both in the 2050s and at the end of the century.

#### Relative sea-level rise

In addition to changes in the mean sea level, an assessment of coastal flooding should consider the contribution of the long-term vertical movement of the land, i.e., local subsidence [28], from either natural or anthropogenic causes. We defined the relative sea-level rise (rSLR) by adding to the regional sea-level rise projections the local natural vertical land movements in each coastal segment using the global model of isostatic adjustment from [29]. To account for the extra subsidence due to natural sediment compaction in deltas, we assumed an additional 2 mm/yr in the most relevant deltas in each region, following the method of [30]. Additional contributions to subsidence due to human action or uplifts due to tsunamigenic events such as the one experienced in Chile in 2010 (e.g., [31]) were not included in this study.

#### Extreme sea levels

To study extreme flooding events, we proceeded in a manner similar to [26] and defined the Total Sea Level (TSL) by linearly aggregating four sea level components: mean sea level, astronomical tide, storm surge and wave setup; using data from previous studies of the region [26, 32]. However, the contribution of tropical storms was not included in the datasets because of insufficient resolution. Historical changes across LAC suggest more frequent extreme sea levels [33–35]. The probability of occurrence of extreme total sea levels in the region notably increased from 1950 to 2008. Historical data show a widening of the statistical distributions toward more frequent and greater extremes overall and on the east coast, the Caribbean and Rio de la Plata in particular [26]. For the future, there is low confidence in regional projections of surges and waves [28]. However, observed changes in extreme sea levels are consistent with mean sea level trends, indicating that future rSLR will further contribute to an increase in the frequency of extreme sea levels [22].

Similar to [26] and [22], we conducted a non-stationary extreme value analysis based on a General Extreme Value (GEV) distribution applied to each historical time series of TSL, which captures the geographical variability in the region. We assumed the 100-year TSL was representative, but the analysis could be easily extended to other return periods. As a first approximation, the future extreme TSL was obtained by uniformly shifting the GEV distribution of the TSL with the projected rSLR. A preliminary analysis of the various drivers (Table 1) showed that there exists significant regional variability across LAC that should be factored in.

**Table 1:** Representative values of contributions to flooding for various timeframes. Regional mean, minimum (Min) and maximum (Max) values indicate the significance of the spatial variability in LAC. rSLR: relative Sea-Level Rise; TSL: Total Sea Level.

Driver (in m)	Present (2011)			2046–2065			2081–2100			
	Mean	Min	Max	Mean	Min	Max	Mean	Min	Max	Comments
rSLR				0.262	0.126	0.385	0.473	0.225	0.712	RCP4.5 including subsidence
rSLR				0.301	0.171	0.425	0.634	0.362	0.886	RCP8.5 including subsidence
ENSO sea level contribution	0.050	-0.034	0.244							1998 event
Extreme total sea level (TSL; 100- year event)	2.687	0.627	7.906	2.813	0.711	7.977				Extrapolation of non-stationary extreme statistics in the historical span (1950–2008)
Subsidence				-0.009	-0.042	0.082	-0.018	-0.085	0.165	Extended into the future linearly. Positive sign denotes subsidence and negative, land uplift

#### Population and economic growth

Population growth was the single socioeconomic driver used in this analysis. We used the Global Rural-Urban Mapping Project data with 1,000 m resolution and referenced to the year 2000 [36]. We considered population size to be a suitable indicator of exposure because the size can be easily translated into exposed assets using estimates of built capital per capita [15]. Using past LAC population growth rates from the Economic Commission for Latin America and the Caribbean (ECLAC) [16] (i.e., an annual increase of 1.24% on average), the values of population size were extrapolated to 2011 to establish a common reference with the built capital. This approach is conservative considering the past coastal and urban agglomeration in the region. Future values of the population size were obtained using LAC projections given in [16]. We note that no assumptions were made in the distributions and the concentrations of the populations; i.e., we assumed national rates to be representative of coastal areas and did not account for additional population concentration in the coastal areas.

We calculated the ratio of built capital (i.e., assets) per capita to gross domestic product (GDP) per capita, measured both in constant 2005 USD and constant 2011 purchasing power parity (PPP) international dollars, using World Bank data and following the procedure described in [15]. Specifically, the input information included the produced capital per capita, the GDP per capita and the GDP growth. We first calculated coefficients for each country to transform between units, and we estimated the built capital throughout the region (see S1 Appendix for further details). The mean value of the built capital per capita for the region was 2.5, which is consistent with a mean estimate of 2.7 obtained by [15] in a global assessment. However, our approach provides nondimensional ratios for each country and therefore allows a comparison of different pathways among nations.

#### Set of scenarios

Various combinations of flooding and socio-economic drivers and timeframes were used to construct a set of scenarios, which are presented in Table 2.

**Table 2:** Scenarios of drivers and timeframes. rSLR: relative sea-level rise; RCP: Representative Concentration Pathway.

DRIVERS OF RISK	Conditions	Timeframe	Inundation by rSLR						Interannual variability: El Niño induced sea levels			rSLR with interannual variability						Extremes		rSLR with extremes			
rSLR	RCP4.5	2050	X	X								X	X										
	RCP4.5	2090			X	X								X	X					X		X	
	RCP8.5	2090					X	X								X	X				X		X
El Niño	1998 event	2010							X	X	X												
	1998 event	2050										X	X										
	1998 event	2090												X	X	X	X						
Extreme Total Sea Level	100 year event, in 2010	2010																X		X	X	X	X
	100 year event, extrapolate to 2050	2050																	X				
	-	2090																					
Population	ECLAC	2010	X		X		X		X			X		X		X		X		X		X	
	ECLAC projections	2050		X						X			X						X				
	ECLAC projections	2090				X		X			X				X		X				X		X

### 2.2. Geospatial analysis of exposure

Populations and land areas exposed to inundation and flooding were computed by discretizing the LAC coastlines into 5 km segments, 14,500 in total. This spatial discretization was obtained using three criteria:

1. A working scale of 5 km of coastline was chosen as a compromise between the resolution of the information available and the spatial scope of the study.
2. The polygons extend 20 km inland from the shoreline, a limit that covers most low-lying areas. However, this approximation may not fully cover deltas and estuaries, where low-lying areas extend farther inland.
3. The polygons were compared with topological rules to ensure accuracy in complex areas and manually corrected if necessary.

Population size and land area were estimated at various topographic elevations for each 5 km segment. The geospatial evaluation process is described in the S2 Appendix in more detail. S1 Fig shows two examples of 5 km geospatial segments for two different types of coastline.

We used elevation data obtained from the Shuttle radar topography mission, which provides a resolution of 90 m horizontally and 1 m vertically [37]. The combination of the radar topography data with the GRUMP (Global Rural-Urban Mapping Project) population dataset produces a conservative estimate when compared with other global data [7, 38]. The zero topographical reference was corrected to the high tide (the 90^th^ percentile of the astronomical tide) in each segment. Our regional values of population size and land area in low-lying locations (below an elevation of 10 m) were consistent with previous estimates for the region [39, 13].

Table 3 provides some information on the geospatial discretization for several countries and shows how the approach accounts for a large proportion of the national territories of the island nations (e.g., Bahamas). The approach accounts for the proportion of low-lying terrain to the total land area of a country. For example, Turks and Caicos (TCA) and the US Virgin Islands (VIR) are both completely covered by the 5 km segments (i.e., the entire land area of the nation is covered by the coastal delimitation criteria). However, TCA consists largely of low-lying terrain, whereas only a small fraction (12.1%) of VIR is below 10 m. Similarly, a large part (79%) of Puerto Rico is near the shore, but less than 10% of the national territory is low-lying (i.e., below 10 m). In contrast, large countries such as Mexico have a low proportion of land near the coast with respect to their total territory, but these are some of the most exposed countries in terms of absolute values. These considerations are relevant for coastal hazards and exposure assessments and are represented in our final estimates of exposure.

**Table 3:** Values of land area covered by the discretization segments for several countries.

Country	Percentage of coastal areas below 10 m (*)	Percentage of land covered by the discretization segments (*)
Bahamas	79.1	88.9
Cayman Islands	72.5	97.2
Mexico	2.4	8.5
Puerto Rico	9.7	78.9
Anguilla	29.5	96.3
Trinidad and Tobago	7.9	90.3
Turks and Caicos Islands	99.6	100.0
Virgin Islands (US)	12.1	100.0

(*) denotes that the value is with respect to the total land area of a country.

Based on the estimates of exposure for the various scenarios, we were able to build spatial maps that identify critical zones and demonstrate the variability in exposure across the region. In the maps used to identify critical zones, the results for the various scenarios and combinations of drivers are presented in 50-km blocks rather than the 5 km coastal segments for clarity. The spatial results were classified into four exposure levels, ‘Low,’ ‘Medium,’ ‘High’ and ‘Very High,’ using the Jenks natural breaks optimization algorithm [39] and certain minimum thresholds (these are specified in the figure labels). From the analysis of the exposure level maps, we identified and assessed the most critical areas and significant drivers of risk in LAC.

## Results and Discussion

### 3.1. Inundation by Sea-Level Rise

**Fig 1:**
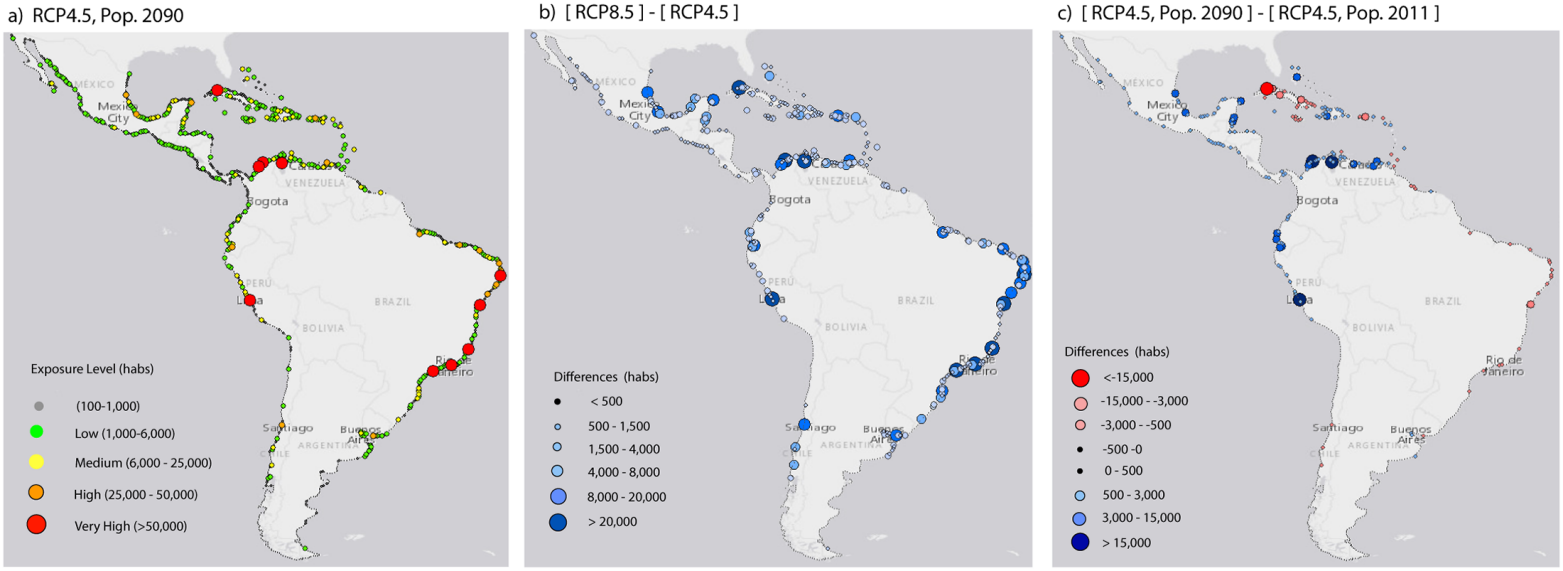
Exposure of population to rSLR scenarios. (a) Exposure levels for population projected to 2090 and rSLR for RCP4.5 (b) Difference between RCPs (c) Difference in exposure to the RCP4.5 sea levels for population projected in 2090 and reference levels (year 2011).

Fig 1 depicts the spatial variation of populations exposed to the rSLR projections by the end of the century. The patterns indicate a surprising spatial consistency between the two RCPs. Fig 1(b) presents the differences between the scenarios. Although a larger number of inhabitants will be affected by the higher sea levels resulting from RCP8.5 than those resulting from RCP4.5, the critical areas most exposed can be consistently identified, and the debate can be narrowed to the number of inhabitants exposed. The areas most exposed are certain locations in the Caribbean islands, the east coasts at latitudes from 0° to 30°S and certain locations on the Pacific coasts of Peru and Ecuador. The differences in the effects of the two RCPs are more pronounced in those areas. Fig 1(c) isolates the effect of the population driver by representing the difference between the exposed levels in 2090 and the reference of 2011. Population growth is a greater driver in the southern Caribbean and the tropical Pacific, whereas estimates of lower population sizes in the future will reduce the exposure in Cuba and other Caribbean islands. With the exception of a few critical zones, we can conclude that overall the rSLR projections have a greater effect than the population change.

**Fig 2:**
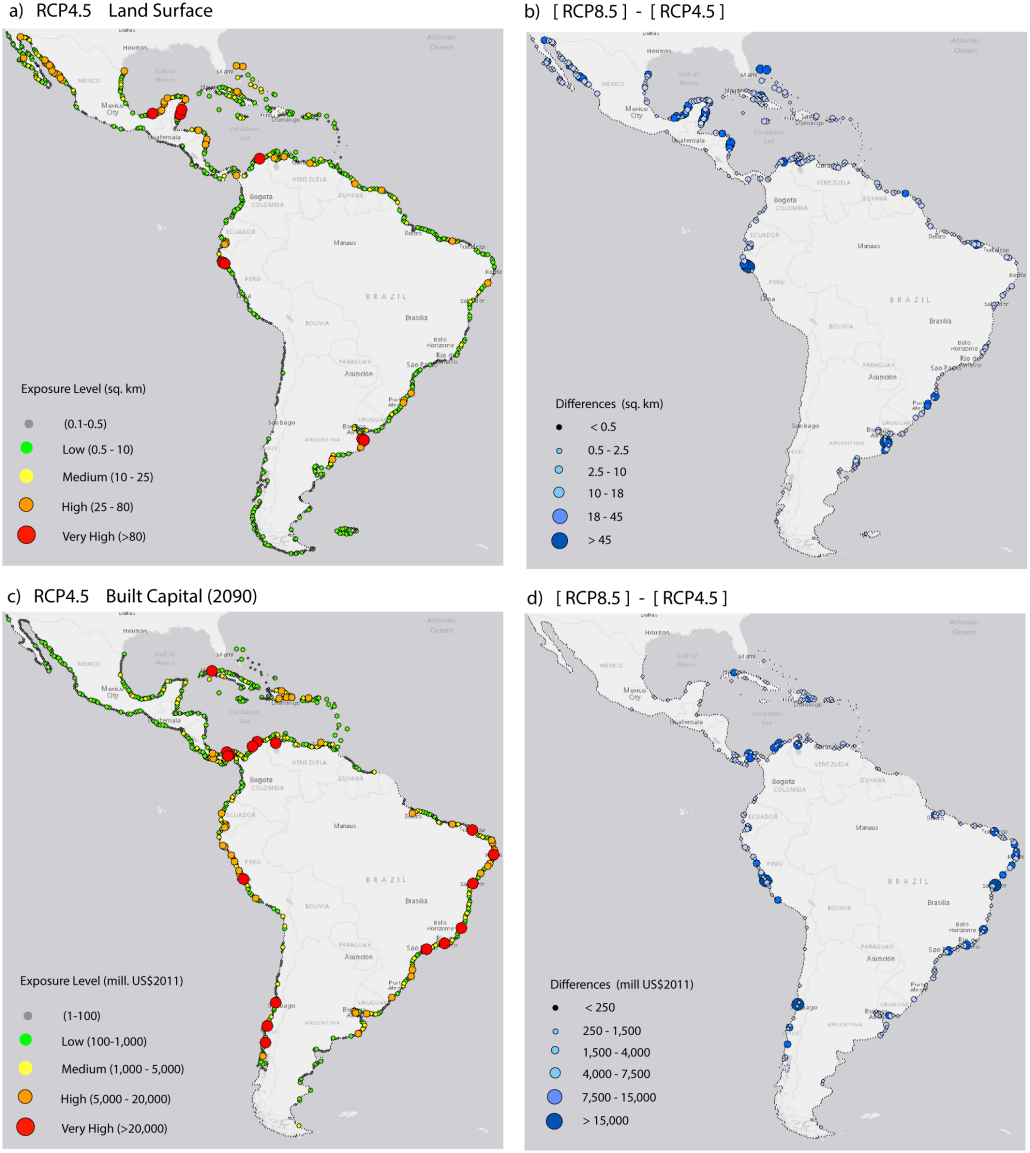
Exposure of land surface (upper panels) and Built Capital (lower panels) to rSLR scenarios. (a) Exposure levels for land inundated by rSLR for RCP4.5 (b) Difference between RCPs for land inundated (c) Exposure levels for built capital inundated by rSLR for RCP4.5 (d) Difference between RCPs for land inundated.

The largest land loss (Fig 2) does not occur in the areas most exposed in terms of either population size or built capital (i.e., land not highly populated or developed currently). In terms of land loss, the most critical areas are the Yucatan peninsula, the southern Caribbean, central Cuba, small islands in the northern Caribbean, small areas at intermediate latitudes on the Atlantic coast (e.g., Paraiba in Brazil), Mar del Plata and Bahia Blanca (Argentina), and northwestern Mexico, northern Peru and Ecuador on the Pacific side. Built capital is closely correlated with population distribution, and the most exposed locations are concentrated in urban areas in the southern Caribbean and the tropical Atlantic and Pacific coasts.

The differences in the effects of the two RCPs on built capital are similar to those for population. The differences are limited to the amounts of land and built capital, but the most critical areas are consistent for both variables and all scenarios.

These results indicate that the exposed areas at the greatest risk in terms of population and built capital do not generally coincide with those areas with the highest potential land loss. This conclusion is not surprising because of the population concentrations in urban and coastal areas in the region [3, 16]. These results also suggest that adaptation responses and policies may depend on whether the goal is to protect land or the inhabitants. Furthermore, these actions and policies may depend on the location.

Table 4 provides regional aggregates of exposure to rSLR. Regionally, more than 4 million inhabitants and 7,000 km^2^ will be at elevations below the highest projected sea level for the end of the century in LAC (assuming the rSLR projection for RCP8.5). For the milder RCP, the values are 3 million inhabitants and 5,300 km^2^. These predictions underestimate the land loss around large estuaries and deltas because of the approach to segment the coastal area (see previous section). The amount of affected built capital increases from 112–150 billion USD at the present exposure to 1,500–2,000 billion USD in projected assets. However, at mid-century the exposed population will number 2 million, a 17% increase because of population growth. The contribution of the population expansion with respect to sea-level rise will be more limited by the end of the century because the population is expected to decrease in the second half of the century.

**Table 4:** Regional exposure to inundation and flooding for various scenarios. rSLR: Relative Sea-Level Rise; TSL: Total Sea Level; the TSL_100_ corresponds to the 100-year event; [2011] refers to present exposure values; [Projected] refers to values projected into the future using population projections for the region.

Driver		Exposure				
		Land surface (x1,000 km^2^)	Population (mill. People)		Built Capital (x1,000 mill. 2011USD)	
			In 2011 values	Projected	In 2011 values	Projected
Inundation	rSLR_2050_	3.16	1.70	2.01	67.0	178.2
	rSLR_2090_ RCP4.5	5.33	2.85	3.05	112.5	1,529.8
	rSLR_2090_ RCP8.5	7.09	3.82	4.09	150.4	2,056.7
Flooding from extremes	TSL_100, 2011_	33.87	7.53	8.89 (*2050*) 8.03 (*2090*)	299.1	819.4 (*2050*) 4,227.8 (*2090*)
	TSL_100, 2050_	42.62	8.40	9.92	334.4	926.6
	TSL_100, 2011_ + rSLR_2090_RCP4.5	44.12	8.42	9.00	335.4	4,825.0
	TSL_100, 2011_ + rSLR_2090_RCP8.5	48.06	8.72	9.32	347.7	5,036.4

### 3.2. Effects of El Niño events with future sea levels

The slowly rising mean sea level is not the only factor driving coastal inundation. El Niño can significantly influence sea levels over the entire tropical west coast of LAC. Previous El Niño events have caused temporary increases similar in magnitude to those caused by the rSLR throughout the last six decades [26]. We now focus on comparing the El Niño-induced sea level changes and the rSLR projections.

The number of inhabitants affected by El Niño events alone and in addition to future rSLR will be substantial in six countries (Peru, Ecuador, Panama, El Salvador, Costa Rica and Guatemala) that have experienced the highest increases in sea level from El Niño events in the past (see Table 5). Adding the El Niño 1998 sea level to the mid-century rSLR projections, the exposure would reach 341,000 inhabitants and 7.1 billion USD of 2011 built capital, a threefold increase over El Niño effects alone. These estimates are comparable to the RCP4.5 exposure values by the end of the century. In aggregate values, if the El Niño effects are added to the rSLR projections, between 27% and 36% more of the population will be exposed than with the rise of mean sea level alone.

**Table 5:** Exposure in countries most affected from El-Niño sea levels. Comparison of sea-level rise projections and El Niño 1998 sea levels. The aggregate values correspond to the six countries that have been most affected by El Niño sea levels in the past [26]: Peru, Ecuador, Panama, El Salvador, Costa Rica and Guatemala. [N98]: mean sea level induced by El Niño 1998 event; [rSLR_2050_]: relative sea-level rise for mid-century; [rSLR_2090_ RCP4.5]: scenario of relative sea-level rise for the end of the century corresponding to RCP4.5; [rSLR_2090_ RCP8.5]: scenario of relative sea-level rise for the end of the century corresponding to RCP8.5.

Driver	Land surface (km^2^)	Population (x1e5 people)		Built Capital (x1,000 mill. 2011USD)	
		In 2011 values	Projected	In 2011 values	Projected
N98	336.4	0.91	1.27 (*2050*)- 1.28 (*2090*)	2.6	14.5 (*2050*)- 148.7 (*2090*)
rSLR_2050_	459.9	1.54	2.14	4.6	27.5
rSLR_2050_+ N_SL_98	796.3	2.45	3.41	7.1	41.9
rSLR_2090_ RCP4.5	774.2	2.59	3.58	7.7	521.6
rSLR_2090_ RCP8.5	1,029.2	3.45	4.78	10.2	698.2
rSLR_2090_ RCP4.5 + N98	1,110.6	3.50	4.86	10.2	670.4
rSLR_2090_ RCP8.5 + N98	1,366.1	4.37	6.06	12.8	846.9

For the six countries most affected by El Niño (see Table 6), an event such as El Niño 1998 would affect between 13% and 38% of the 2011 population levels exposed to the worst SLR projection by the end of the century. However, if we add El Niño 1998 sea levels to the sea levels at mid-century, the figures increase to 58–83%, similar to the values for the RCP4.5 projection by the end of the century, and the values will be even greater in Ecuador and El Salvador. Furthermore, in Ecuador, El Salvador, Costa Rica and Guatemala, an El Niño 1998 event in addition to RCP4.5 sea levels by the end of the century will result in more inhabitants exposed than with the RCP8.5 scenario alone. Moreover, El Niño events atop end of the century sea levels increase the values of population exposed differently across countries, Ecuador showing the largest rise.

**Table 6:** Population exposed under various scenarios for the countries most affected by El Niño sea levels. All values are expressed in percentages relative to the sea-level rise scenario RCP8.5 at the end of the century and the population size in the year 2011. For the aforementioned scenario, values of population size are provided in brackets for the reference year. [N98]: Sea level associated with El Niño 1998; [rSLR]: projections of relative sea-level rise; [RCP4.5] and [RCP8.5] refer to rSLR projections for the two concentration scenarios. The last column, the ratio of low-lying population, refers to the proportion of the population occupying land at elevations below 1 m with respect to the population occupying land at elevations below 10 m, as calculated at the 2011 population levels, and is used to indicate the susceptibility of low-lying areas within countries.

COUNTRY	Mid—Century			End of Century				
				2011 population)[rSLR]		[rSLR + N98]		
	N98	rSLR	rSLR+ N98	RCP4.5	RCP8.5	RCP4.5	RCP8.5	Ratio of low-lying population (calculated for
	VALUES FOR POPULATION IN 2011							
Peru	23%	45%	67%	75%	100% (202,787)	98%	123%	51%
Ecuador	38%	45%	83%	75%	100% (81,100)	113%	138%	22%
Panama	13%	44%	58%	74%	100% (25,527)	88%	113%	13%
El Salvador	34%	45%	79%	75%	100% (16,276)	109%	134%	15%
Costa Rica	27%	45%	71%	75%	100% (10,991)	102%	127%	19%
Guatemala	26%	45%	71%	75%	100% (8,282)	101%	126%	6%
	In projected values of population to 2050			In projected values of population to 2090				
Peru	31%	59%	90%	96%	128%	125%	157%	-
Ecuador	57%	67%	124%	120%	159%	180%	219%	-
Panama	20%	68%	88%	118%	159%	139%	180%	-
El Salvador	42%	54%	96%	89%	118%	129%	159%	-
Costa Rica	33%	56%	89%	81%	108%	110%	137%	-
Guatemala	48%	83%	131%	162%	216%	218%	272%	-

Population growth will increase the exposure further, by 59% in Ecuador and Panama and 116% in Guatemala for RCP8.5. Furthermore, if we define the ratio of low-lying population as the fraction of the population occupying land at elevations below 1 m relative to those occupying elevations below 10 meters above the present mean sea level, we find that many of these countries have moderate ratios (Table 6). This metric may serve as an indicator of further indirect effects. Note that Peru in particular has a very high ratio of 51%, whereas the ratio for Guatemala is only 6%.

**Fig 3:**
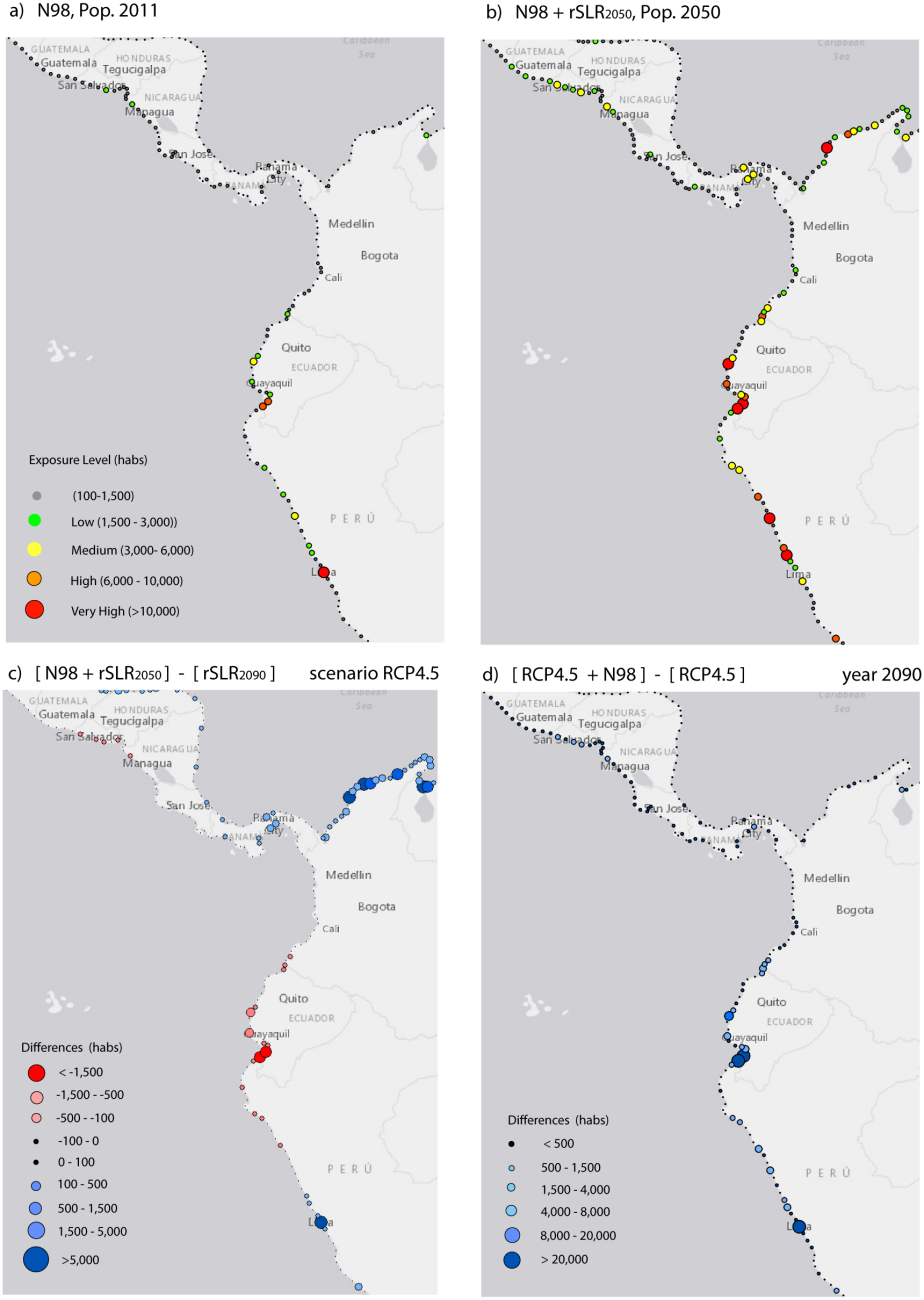
Population exposed to El-Niño scenarios. (a) Sea level induced by the El-Niño-1998 event with population in 2011 (b) Sea level induced by the El-Niño-1998 event at the mid-century, including rSLR (c) Difference of exposure between El-Niño-1998 by the mid-century and the SLR projection for RCP4.5 (d) Difference of exposure between El-Niño-1998 atop the rSLR projection for RCP4.5 and the rSLR projection alone. [N98]: El-Niño-1998 event induced mean sea level; [rSLRyy]: relative sea-level rise for year ‘yy’; [RCP4.5]: scenario of rSLR for the end of the century corresponding to the RCP4.5.

Fig 3 shows the threat that El Niño combined with rSLR could represent in LAC for present and future populations. El Niño is already one of the main drivers of flooding on the tropical Pacific coast, but if El Niño events comparable to that of 1998 occur at the mid-century, even with moderate rSLR values (i.e., small differences between projections by mid-century), the exposure values are nearly the same as RCP4.5 estimates for the end of the century (Fig 3(c)). These results indicate that the effects of El Niño on sea level and coastal exposure have been grossly underestimated. El Niño events should be considered more carefully in coastal zone management in risk-prone countries, particularly given the synergetic effects of El Niño and higher sea levels from climate change. Indeed, when considering an El Niño 1998 event in addition to rSLR projections (Fig 3(d)), the exposed population size increases significantly at specific coastal locations, particularly in Ecuador and Peru.

It has to be noted, however, that effects from El Niño sea levels are not permanent and last only several months, and thus the management responses will be different from those for rSLR despite the comparable exposure levels. Nevertheless, inter-annual variations in sea levels can occur in only a few months, and thus anticipation and planning for the mid-century timeframe for a variety of combinations of rSLR and inter-annual variations should merit proactive measures implemented sooner than would be the case for rSLR alone.

### 3.3. Coastal flooding events

**Fig 4:**
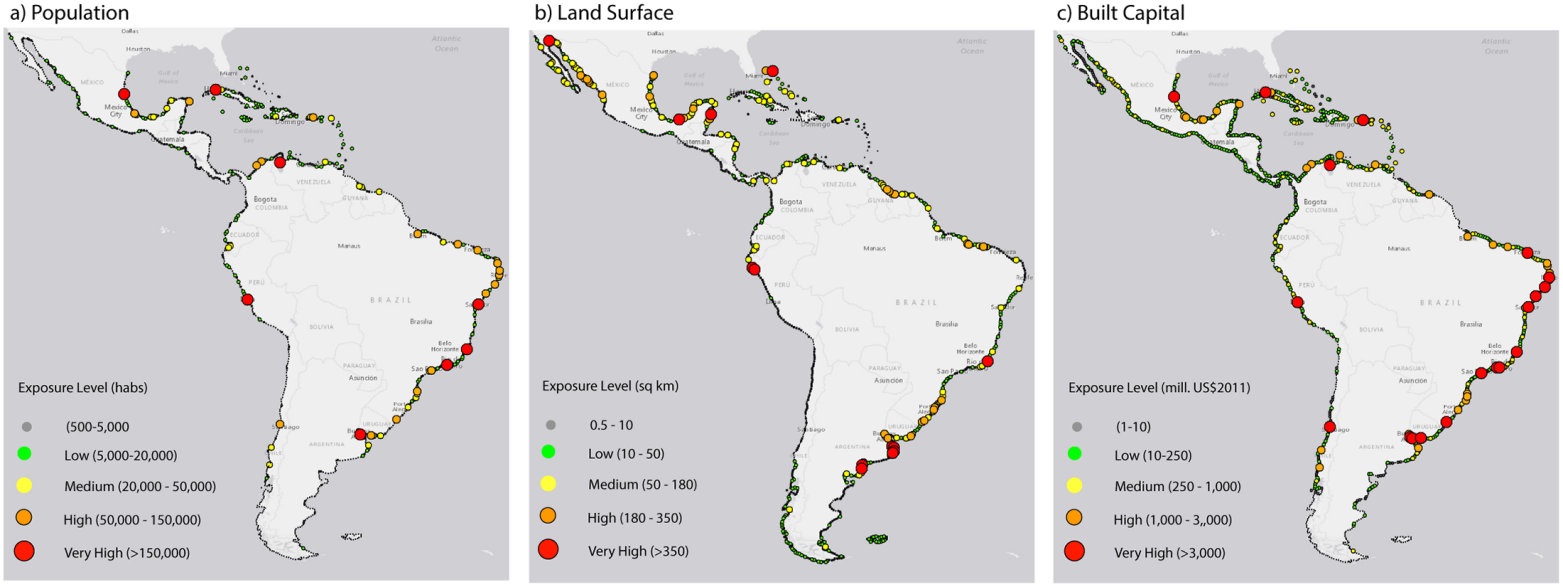
Flooding exposure from present 100-yr extreme sea level. (a) Population; (b) land surface and (c) built capital at 2011 reference values.

The population, land and built capital exposed to coastal flooding from extra-tropical storms (for the 100-year extra-tropical extreme sea level) vary greatly throughout the region (Fig 4). The most exposed populated centers do not coincide with the most exposed land as found for inundation from rSLR. Furthermore, some locations of hotspots differ to those with highest exposure to rSLR. Region-wide (see Table 4), more than 7.5 million inhabitants and 299 billion USD in built capital are presently exposed to flooding. This estimate increases to 8.9 million inhabitants and more than 819 billion USD by 2050 considering solely the effect of population growth. Exposure will decrease to approximately 8 million inhabitants in the second half of the century assuming no elevated sea level. The projected decrease in population size for the second part of the century partly offsets the increase in the hazard term. Nevertheless, the rSLR for the RCP4.5 and RCP8.5 scenarios will affect 8.4 and 8.7 million inhabitants, respectively, assuming year 2011 populations, and these values will fall between 9 and 9.3 million by the end of the century if the population size, rSLR and the present extreme sea levels are factored in.

As we showed for El Niño, the situation at mid-century is already comparable in exposure levels to the end-of-the-century scenarios. If we assume that the extreme sea levels continue to change at the historical rate, the mid-century situation yields the same exposure as the end of the century with rSLR and no change in storm activity (i.e., current flooding levels on top of rSLR projections). Ignoring the population size, the extrapolated coastal flooding by mid-century will affect the same levels of population and built capital as the present levels of storm activity combined with the projected rSLR.

Interestingly, when the region-wide exposure is expressed in terms of annual expected risk from the present 100-year flooding level (multiplying the probability of occurrence of the hazard by the exposure levels), more frequent extremes imply that the risk for mid-century is more than 5 times greater, even with no increase in population (Table 7). This value was calculated considering the increasing probability of extreme sea levels observed in the recent past [26].

**Table 7:** Flooding risk for populations expressed in terms of annual expected risk. rSLR and TSL denote sea-level rise and potential flooding levels, respectively. [1/100] refers to the probability of occurrence of the 100-year extreme sea level in 2011 as a reference (present); [T(2050)] denotes the return period associated with the 100-year 2011 sea level calculated in year 2050 from the extrapolated long-term changes in the extremes from the historical record (1950–2008); [RCP4.5] and [RCP8.5] refer to the two sea level rise projections by the end of the century. Percentage values are expressed with respect to the 100-year event in the reference year, 2011.

Annual Average Risk: [Exposure x Prob. Exceedance]					
Sea Level terms		TSL_100, 2011_	rSLR_2050_ + TSL_100, 2011_	RCP4.5+ TSL_100, 2011_	RCP8.5 + TSL_100, 2011_
Probability of exceedance		1/100	1/T(2050)	1/100	1/100
**Exposure values of population (mill. of people/yr)**	**Projected**	-	0.465	0.090	0.093
	**in 2011 values**	0.075	0.393	0.084	0.087
	**Projected**	-	620%	126%	133%
	**in 2011 values**	100%	524%	120%	124%

These results indicate how crucial it is to account for changes in storm activity and the resulting changes in extreme sea levels, both in intensity and frequency. Storminess ultimately defines the extreme flooding levels and will be responsible for an appreciable risk in the near future.

**Fig 5:**
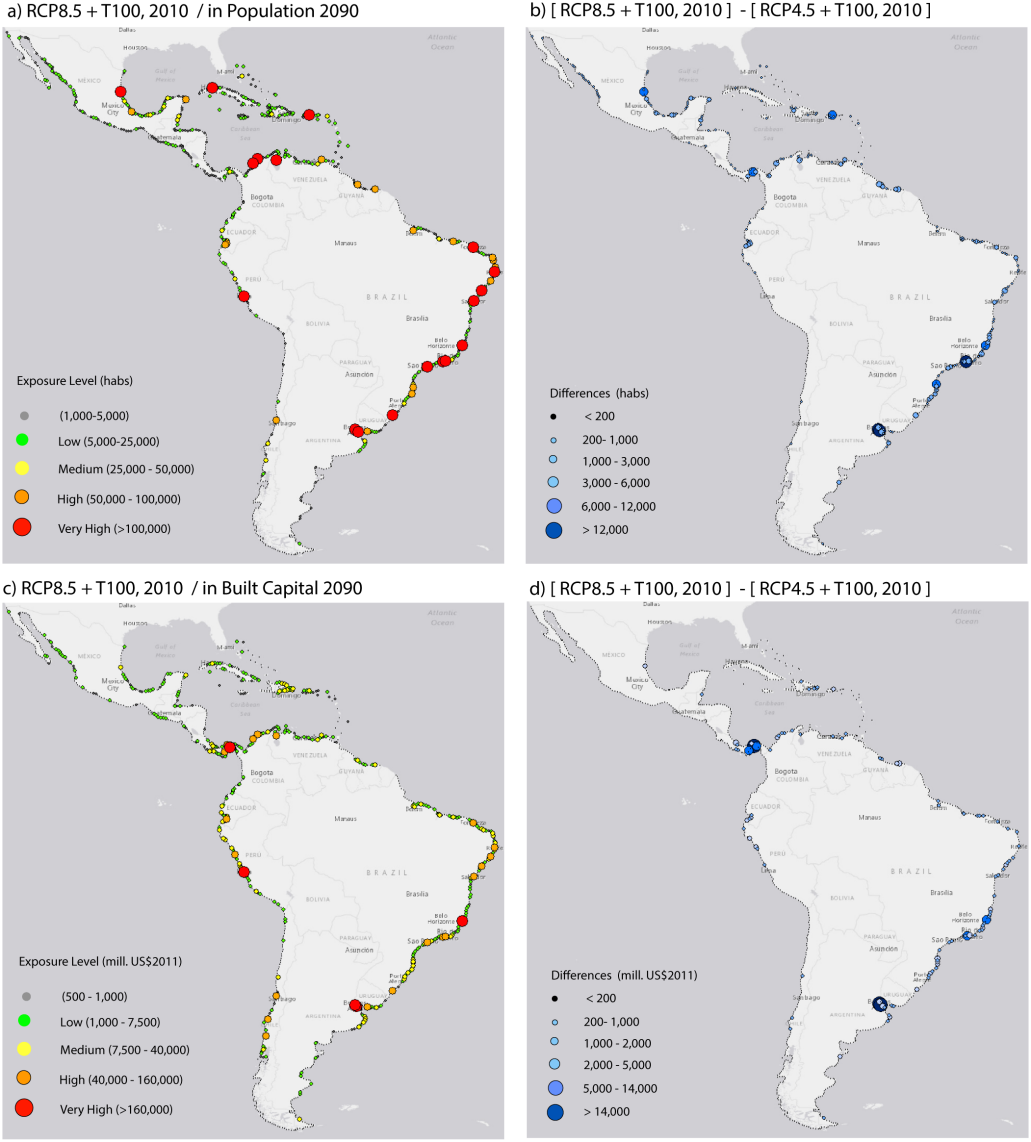
Exposure of population and built capital to future rSLR and present 100-yr extreme sea level. (a) Population exposed to RCP8.5 rSLR and present 100-yr extreme sea level; (b) difference of exposed population between RCPs (c) Built capital exposed to RCP8.5 rSLR and present 100-yr extreme sea level; (d) difference of built capital exposed between RCPs.

To demonstrate the spatial variability of exposure to extreme sea levels, Fig 5 shows the present 100-year flooding event in addition to the rSLR projections. Populations most exposed are found in Central America (e.g., Cancun and Tampico), the Caribbean islands (e.g., San Juan and La Habana), the east coast (e.g., Fortaleza, Natal, Recife, Rio de Janeiro, Florinapolis, Montevideo and Buenos Aires) and certain locations along the west coast (La Libertad and Guayaquil in Ecuador, Lima in Peru, and Valparaiso in Chile). Although the built capital is generally correlated with population, some remarkable differences were found in the Caribbean and the southwestern coastal areas.

Differences in exposure resulting from the two RCPs (right panels in Fig 5) are more noticeable in the southern Atlantic coast, followed by certain Caribbean population centers. Differences in built capital are most prominent in Mesoamerica and Buenos Aires and slightly less in southern Brazil. Nevertheless, and as we found for the rSLR projections, the most exposed locations can be consistently identified across scenarios and drivers, which can be useful for prioritizing affected areas.

These results are consistent with those of previous reports and observations in the region. For example, a global analysis of the largest coastal port cities similarly identified Guayaquil as particularly at risk [15]. Our study identifies additional locations at-risk, primarily because of the greater spatial definition of the drivers and because the analysis was not restricted to major port cities.

The results suggest that rSLR will be a major driver in the future and will increase future risk, particularly in the tropical region of LAC, which is characterized by low tidal ranges. Tampico and Cancun in Mexico, San Juan in Puerto Rico, Barranquilla and Cartagena in Colombia, Maracaibo and Cumana in Venezuela, Georgetown in Guyana and Paramaribo in Suriname are some examples of high-risk locations in the southern Caribbean, where rSLR poses a significant threat. Furthermore, this area is prone to hurricanes and tropical storms, which will further add to the overall risk. These results are consistent with other studies of the Caribbean, where losses in major port cities will increase the most from SLR in the future [15].

At the southern latitudes, where coastal areas experience the highest tidal ranges, extreme sea levels are the critical driver rather than rSLR [26]. High-risk areas that should consider managing this driver include Buenos Aires, Itajai, Florianopolis, Rio de Janeiro and Grande Vitoria. These findings reinforce the importance of studying the extremes; their spatial variability will be critical in future impact assessments associated with climate change.

Finally, although more inhabitants will be exposed to episodic flooding, the damage will be recurrent and not permanent as for rSLR. The effects will also strongly depend on the physical pathway to the affected area (e.g., coastal barriers). There are important considerations here for coastal management, risk reduction and adaptation. Although certain measures could be implemented at the pace of slowly rising sea levels, protection from storm flooding events requires proactive planning and implementation in areas where the effects of floods could be more acute (Fig 5). The same logic applies to El Niño and other inter-annual events, as encountered previously in the tropical Pacific area.

## Conclusions

This study compared various drivers that affect present and future exposure to coastal flooding and inundation, addressing a lack of information on Latin America and the Caribbean (LAC). We compared regional relative sea-level rise (rSLR) projections for the end of the century, inter-annual variations in sea levels induced by El Niño and extreme extra-tropical flooding levels. We identified locations of concern and provided timeframes and exposure levels that will require further action and planning. With between 3 and 4 million inhabitants exposed by the end of the century from rSLR alone, we found that the areas most at risk could be consistently identified across RCPs, which could be useful in choosing adaptation actions.

Most notably, we showed that the contributions from El Niño events substantially raise the threat in most Pacific-coast countries of the region and sooner than previously anticipated. Exposure levels from El Niño events in addition to rSLR by mid-century are comparable to the end-of-century estimates for rSLR alone. Furthermore, when El Niño-induced sea level changes are added to rSLR projections resulting from climate change the exposed population increases significantly, particularly along the coastlines of Ecuador and Peru.

Currently, 7.5 million inhabitants and 299 billion USD in built capital are exposed to flooding from a 100-year event in LAC without considering hurricanes. With both extreme sea levels and populations increasing, this exposure will increase to 8.8 million inhabitants by mid-century. However, if we consider the historical trend of storm activity, this value could be closer to 9.9 million inhabitants in the same timeframe. This effect will be equivalent to the projected rSLR for the end of the century with no additional change in the present extreme sea levels. Region-wide, exposed built capital will also increase greatly for all of the scenarios considered.

Expectations of higher sea levels, coastal development and population growth will lead to globally higher risks from coastal hazards [4, 13, 15, 40–42]. This study examined the case for LAC. The present analysis considered both spatial variations in the hazards and socio-economic development within countries that resulted in two sources of variability in identifying the exposure distribution throughout the region. However, local spatial variations will require an additional level of scrutiny for areas at risk currently and in the future. It is likely that the high-risk zones identified here will be at greater risk given the historical trends in the region toward urban development [3]. This analysis did not consider future development, actions or strategies for adaptation. Rather, this analysis may be of use to decision makers by identifying areas, factors and timeframes of concern.

As we demonstrated, significant amounts of land, population and built capital will be exposed to rising sea levels in LAC, and the timeframes could be shorter than previously anticipated when we account for drivers other than rSLR. We hope that the results and conclusions from this study might lead to more sustainable and informed coastal development in LAC.

## Supporting Information

S1 Appendix Built Capital valuation. (DOC)

S2 Appendix Geospatial analysis of exposure variables. (DOC)

S1 Fig Geospatial units for the study. Representative polygons of 5 km of coastline, covering 20 km landwards and 10 km seawards. Exposure variables are geo-processed at 90 m at each unit and results aggregated to 50 km segments for showing and analyzing results. Source of imagery for basemap: Esri, DigitalGlobe, GeoEye, Earthstar Geographics, CNES/Airbus DS, USDA, USGS, AEX, Getmapping, Aerogrid, IGN, IGP, swisstopo, and the GIS User Community.
(DOC)

S1 Table Outline of information and data sources for estimating the Built Capital. (DOC)
