# Supplementary material for: Effects of Climate Change on Exposure to Coastal Flooding in Latin America and the Caribbean
Source: PLoS One. 2015 Jul 15;10(7):e0133409. doi: 10.1371/journal.pone.0133409 (PMC4503776; doi:10.1371/journal.pone.0133409)
Supplement: S1 Appendix — (DOC) [file pone.0133409.s001.doc]

**S1 Appendix. Built Capital valuation**

**1. Theoretical description**

As Hallegate et al (2013) found, there exists a robust relation between Built Capital per capita (a component of total wealth) and the Gros Domestic Product (GDP) per capita that can be formulate as follows:


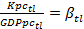
 (1)

where *t* denotes period of time and *l* denotes a particular country.

As a first estimate, we assume that
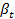
 is stationary, which implies constant rates of growth for both variables. The capital stock per capita (*Kpc*) can be expressed as:


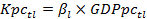
 (2)

Therefore, from the exposed population affected by an impact,
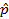
, the estimated total capital stock affected is
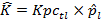
, and for a certain scenario of GDP, population and climate, the total capital exposed in country *l* would be:


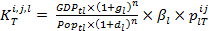
 (3)

where:

*t* and *T* are reference and target year

*i* determines the demographic scenario

*J* defines the climatic scenario

*l* defines the country

*g_l_* and *dl* define the annual rate of growth for GDP and population (%) for the country

*n d*efines the number of time intervals

*GDP_tl_* stands for Gross Domestic Product for the country *l* and a time period *t*

*Pop_tl_* stands for Population at the year *t* at country *l*


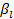
 defines the ratio of per capital and GDP for the country, previously estimated from Built Capital and population data.

*P_Tl_^ij^* is the affected population under the *i* demographic scenario, the *j* climatic scenario, the country denoted by *l* and the target period *T*

This formula can be applied in three different approaches that are described below.

**Case A) Constant growth for both GDP and Population**

If we assume a constant GDP rate of growth, *g*, and a constant population rate of growth, *d*, from the present value of GDP and Population and the country specific
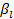
, we can calculate the per capita stock of capital as follows:


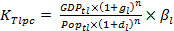
 (4)

And the total affected capital would be:


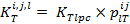
 (5)

However, this formulation relies on the internal consistency between the constant rate of population growth
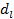
and the externally defined demographic scenario *I,* used to estimate the future population affected by flooding. Consequently the formulation is not generally applicable although valid for specific cases.

**Case B) Projection of Population and a Constant growth for GDP**

If we only predict our scenario for per capita GDP with a constant rate of growth, g_l_, thus avoiding the restriction of constant population growth and using only initial and target population, the demographic rate growth could be substituted by:


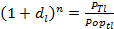
 (6)

And the general expression transforms into:


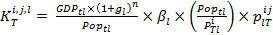
 (7)

The per capita stock of capital at constant population is:


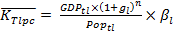
 (8)

And the final expression can be expressed in a compact form as:


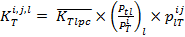
 (9)

Where we multiply the per capita capital by a scale factor correcting its value due to projected final population and then apply the result to the affected population.

An interesting conclusion emerges if we study the flooding of the total population making
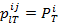
, and going back to the previous expression


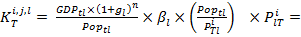
 (10)


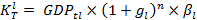


In such a case the total affected capital does not depend on demographic values but on its initial value, its growth projection and the
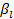
 coefficient of the country.

In our case study, the average GDP pc for Latin America and Caribbean measured in international PPP constant 2011$ is 14,130$, the ratio of present to projected population is 0.83 for 2050 and 0.92 for year 2090 and the average growth observed for GDP is 2.85%. Given a beta value of 2.501, we obtain a per capita built capital of 87,726 $. For a flooded population of 7.53 million persons, or equivalently, 35 bill$ this represents an amount of 660 billion in 2050 and 2,253 for 2090. Hence from 2050 to 2090 the total flooded capital jumps by 3.41.

**Case C) Projected scenarios for both GDP and population**

If we could get estimates for *GDP_Tl_* and *Pop_Tl_* at a certain timeframe, then the problem is reduced to the simple form:


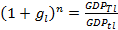
 (11)


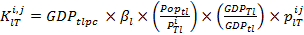
 (12)

And since
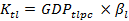
 we obtain initial per capita capital that can be substitute in the formula


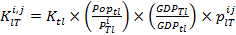
 (13)

That makes the affected capital dependent on flooded population, the initial value of stock of capital and the two ratios of GDP and Population between timeframes.

**2. Application for Latin America and the Caribbean**

We estimate the mean Built Capital per capita from the distribution of population using the national accounts and following the method described in Hallegatte et al (2013). We build the ratio Built Capital pc to Gross Domestic Product (GDP) per capita measured both in constant 2005 USD and Purchasing Parity Power (PPP) Constant 2011 international USD. The ratio is built on a country basis that allows comparison within the region. Based on the World Bank national data for Latin America and the Caribbean, we obtain an average value of 2.5, which is in close agreement with the global value of 2.7 obtained by Hallegate et al (2013).

The GDP per capita is projected into the future considering the minimum historical GDP growth in the last 5, 10, 15 and 20 years. A minimum threshold of 0.5% is set for the GDP per capita growth rate. Since the Built Capital per capita is computed for a constant population, the projected value for future total wealth per capita cannot be directly applied as capital for future flooded population projections. Therefore, we scale with projected population variations to adjust for future demographic pathways.

The data and sources used for the analysis are outlined in S1 Table.

| Data | Units | Description | Original Source |
| --- | --- | --- | --- |
| Produced Capital per capita | 2005usd | Total and per capita wealth of nations data bank | World Bank, 2010 |
| GDP per capita | Current Us$ | Data Bank | World Bank, 2010 |
|  | 2005 Us$ |  | World Bank, 2010 |
|  | PPP (constant 2011 international $) |  | World Bank, 2010 |
| GDP growth | (annual %) | Data Bank | World Bank, 2010 |

Table S1. Outline of information and data sources for estimating the Built Capital.

**3. General discussion of assumptions**

The analysis described above relies strongly on a main assumption: the
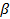
 parameter is defined as a country characteristic and stationary in time. Since we assume that
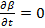
, the capital stock per capita grows at the same rate than GDPpc. This assumption is plausible as far as growth in capital stock is the result of temporal integration of investment and they are a fraction of GDP.

The consequence of this assumption is that for a given period *t* and given an average productivity of capital *r* (stationary in time) and a total capital stock *K*, we can express GDP as
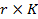
 and hence the parameter can be expressed as:


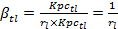
 (14)

Consequently, the average productivity of capital remains constant throughout the whole century.

**Additional References**

*World Bank, 2010 The Changing Wealth of Nations: Measuring Sustainable Development in the New Millennium.*

*Hallegatte, S., Green, C., Nicholls, R.J., Corfee-Morlot, J. 2013. Future flood losses in major coastal cities. Nature Climate Change. Doi: 10.1038/NCLIMATE1979. See Annex in Supplementary Online Material*
