# Supplementary material for: Effects of Climate Change on Exposure to Coastal Flooding in Latin America and the Caribbean
Source: PLoS One. 2015 Jul 15;10(7):e0133409. doi: 10.1371/journal.pone.0133409 (PMC4503776; doi:10.1371/journal.pone.0133409)
Supplement: S2 Appendix — (DOC) [file pone.0133409.s002.doc]

**S2 Appendix. Geospatial analysis of exposure variables**

S1 Fig shows a few of the geospatial units that resulted from the geospatial discretization of the region and where the land surface and population are processed by topographic levels.

The geospatial process at each of such units counts several steps (in “ArcGis” ©, extensions “Spatial Analyst” and “3D Analyst”):

- Delimitate the information to the coastal strip and re-project all the data layers to a common projection that maintains distances and area;
- define the general coastline;
- create the analysis units (polygons representative of a certain area of land and sea) ;
- obtain the flooding masks from 1 to 10 m of elevation at each study unit, keeping the hydraulic connectivity with the sea;
- calculate the area and population affected for each variable at each elevation.
