# Supplementary material for: Effects of Climate Change on Exposure to Coastal Flooding in Latin America and the Caribbean
Source: PLoS One. 2015 Jul 15;10(7):e0133409. doi: 10.1371/journal.pone.0133409 (PMC4503776; doi:10.1371/journal.pone.0133409)
Supplement: S1 Fig — Representative polygons of 5 km of coastline, covering 20 km landwards and 10 km seawards. Exposure variables are geo-processed at 90 m at each unit and results aggregated to 50 km segments for showing and analyzing results. Source of imagery for basemap: Esri, DigitalGlobe, GeoEye, Earthstar Geographics, CNES/Airbus DS, USDA, USGS, AEX, Getmapping, Aerogrid, IGN, IGP, swisstopo, and the GIS User Community. (DOC) [file pone.0133409.s003.doc]

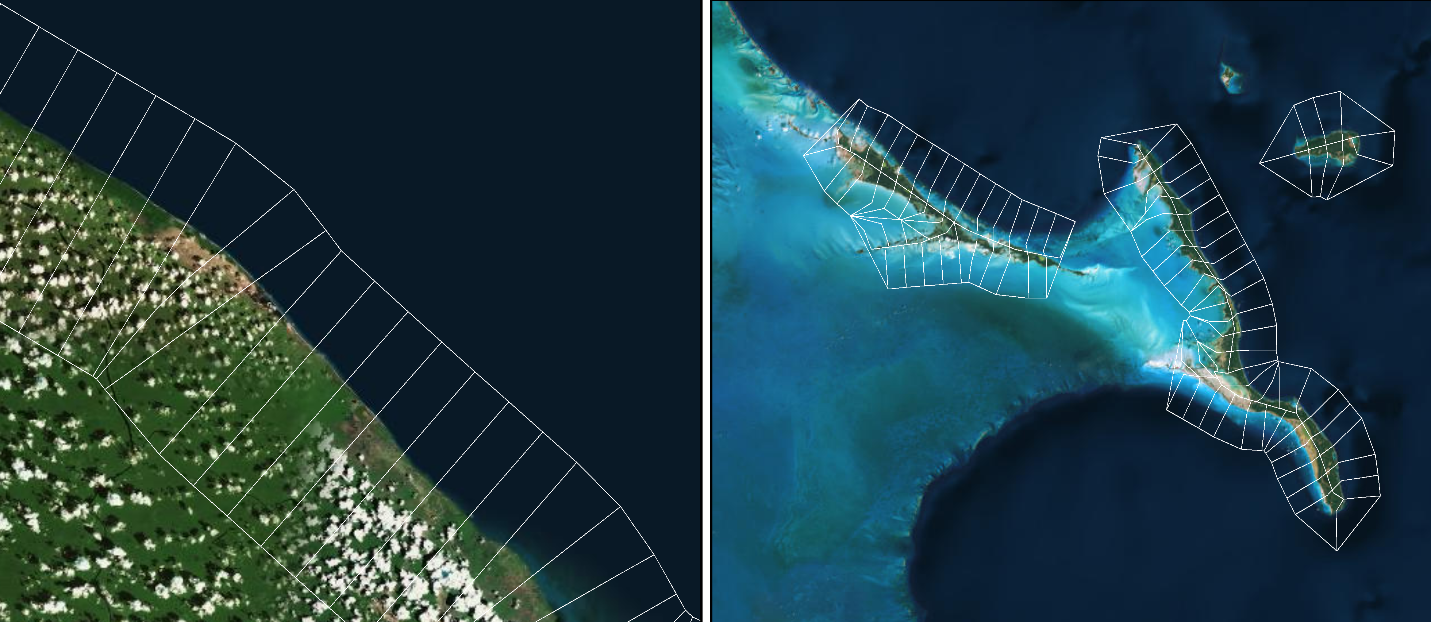


S1 Figure. Geospatial units for the study. Representative polygons of 5 km of coastline, covering 20 km landwards and 10 km seawards. Exposure variables are geo-processed at 90 m at each unit and results aggregated to 50 km segments for showing and analyzing results. Source of imagery for basemap: Esri, DigitalGlobe, GeoEye, Earthstar Geographics, CNES/Airbus DS, USDA, USGS, AEX, Getmapping, Aerogrid, IGN, IGP, swisstopo, and the GIS User Community
