# Supplementary material for: Effects of Climate Change on Exposure to Coastal Flooding in Latin America and the Caribbean
Source: PLoS One. 2015 Jul 15;10(7):e0133409. doi: 10.1371/journal.pone.0133409 (PMC4503776; doi:10.1371/journal.pone.0133409)
Supplement: S1 Table — (DOC) [file pone.0133409.s004.doc]

| Data | Units | Description | Original Source |
| --- | --- | --- | --- |
| Produced Capital per capita | 2005 USD | Total and per capita wealth of nations data bank | World Bank, 2010 |
| GDP per capita | Current USD | Data Bank | World Bank, 2010 |
|  | 2005 USD |  | World Bank, 2010 |
|  | PPP (constant 2011 international $) |  | World Bank, 2010 |
| GDP growth | (annual %) | Data Bank | World Bank, 2010 |

S1 Table. Outline of information and data sources for estimating the Built Capital.
